# The silent struggle after childbirth – barriers and facilitators to help-seeking for postpartum depression and anxiety

**DOI:** 10.1186/s12888-026-08509-9

**Published:** 2026-08-17

**Authors:** Anna Luisa Büssow, Lara Seefeld, Julia Schellong, Susan Garthus-Niegel

**Affiliations:** 1 Institute of Psychology, Faculty of Life Sciences, Humboldt-Universität zu Berlin, Berlin, Germany; 2 Institute and Policlinic of Occupational and Social Medicine, Faculty of Medicine, TUD Dresden University of Technology, Dresden, Germany; 3 Department of Psychology & Neuroscience, Dalhousie University, Halifax, Canada; 4 Department of Psychotherapy and Psychosomatic Medicine, Faculty of Medicine, University Hospital Carl Gustav Carus, TUD Dresden University of Technology, Dresden, Germany; 5 Institute for Systems Medicine, Faculty of Medicine, MSH Medical School Hamburg, Hamburg, Germany; 6 Department of Childhood and Families, Norwegian Institute of Public Health, Oslo, Norway

**Keywords:** Postpartum depression, Postpartum anxiety, Help-seeking intention, Help-seeking behavior, Treatment barriers

## Abstract

**Background:**

The postpartum period makes mothers particularly vulnerable to mental health disorders such as postpartum depression and anxiety. Despite the high prevalence of these disorders, help-seeking for them remains critically low, leaving many women without adequate support. Therefore, the aim of this study was to examine the extent to which different factors are associated with help-seeking intentions and actual help-seeking behaviors for mental health disorders among postpartum women. We also examined how these factors differed between groups of women with symptoms of postpartum depression, postpartum anxiety, symptoms of both disorders, and women without clinical symptoms.

**Methods:**

We used data from a cross-sectional study in Dresden, Germany, with data from *N* = 4,624 women collected via telephone interviews between 2021 and 2024. Multiple linear regression models were used to examine predictors of help-seeking intention (*n* = 4,554) in all the groups, while logistic regression models (*n* = 778) were used to investigate predictors of actual help-seeking behavior among women with symptoms of postpartum depression and/or anxiety.

**Results:**

For the different factors, results varied between help-seeking intention and actual help-seeking behavior. Negative health beliefs were found to have the most negative effect on help-seeking intention for all groups. Help-seeking intentions were lower among women with a migrant background who had symptoms of either postpartum depression or anxiety, among women with lower knowledge of services who were not clinically affected, and lower among women with symptoms of postpartum depression who had not used mental health services before. Recognition of a mental disorder was by far the most important factor associated with higher actual help-seeking behavior for all groups. Higher income and older age were factors associated with higher actual help-seeking behavior among women with symptoms of postpartum depression.

**Conclusions:**

This study identified factors associated with help-seeking for postpartum depression and anxiety. The findings suggest that routine screening for postpartum depression and anxiety to address the lack of recognition of mental disorders, as well as targeted awareness campaigns to support positive health beliefs, are needed and can improve help-seeking. Tailored interventions for at-risk groups, such as low-income or migrant women, could improve postpartum mental health care.

**Supplementary Information:**

The online version contains supplementary material available at https://doi.org/10.1186/s12888-026-08509-9.

## Introduction

While many have an image of a happy mother after childbirth, the reality is that the postpartum period makes women particularly vulnerable to mental disorders [1]. Depression after childbirth, known as postpartum depression (PPD), is considered to be the most common postpartum medical complication [2], with a pooled global prevalence of 14–17.7% [3, 4], yet many postpartum women do not seek help for a mental disorder [5]. The DSM-5 defines PPD as experiencing symptoms of a major depressive episode, such as a depressed mood, loss of interest or energy [6] in the postpartum period. The postpartum period can last until six months after childbirth [7]. Postpartum onset or persistence of a depression after childbirth on average show worse symptoms [8].

Postpartum anxiety (PPA) is even less well known among mothers than PPD and therefore often goes unrecognized [1, 9], and help-seeking remains relatively low. With global prevalence rates ranging from 15.2 to 20.1% [10, 11], PPA is also very common. In the postpartum period, women suffer from different types of anxiety disorders such as generalized anxiety disorder (GAD), panic disorder, specific phobia, or obsessive-compulsive disorder [12]. GAD has the highest prevalence ranging from 4.4 to 10.8% [13], making it particularly relevant to the postpartum period. Since PPA is not mentioned in the DSM-5, the criteria for anxiety disorders are applied to the postpartum period [6]. For example, symptoms of GAD may include worry and difficulty controlling worry [6].

Both PPD and PPA are associated with serious maternal, infant, and family outcomes [14]. Especially untreated PPD [15] is associated with poorer maternal health and can impact the child’s development [16]. Similarly, PPA may negatively affect the child’s development [12], e.g. through maternal bonding. In turn, the entire family system can be affected [17], as maternal PPD is associated with partner and family dissatisfaction and with paternal PPD [18], leading to difficulties in fulfilling parenting responsibilities and meeting child needs [19]. Help-seeking is important to prevent these possible consequences. While many postpartum women experience mental disorders such as PPD or PPA, only a small proportion seeks help, leaving many women to suffer in silence [20]. A systematic review showed that 40–80% of women with PPD or PPA intend to seek help [21], while the prevalence of actual help-seeking behavior for PPD and PPA is much lower (ranging from 4 to 50%; 27–29). To refer to both help-seeking intention and professional, actual help-seeking behavior, this paper uses the term help-seeking as an umbrella term for both concepts, which need to be studied together to provide a more comprehensive understanding of help-seeking.

Possibly, help-seeking differs among women with PPD, PPA, and those affected by both PPD and PPA. Each disorder as well as comorbid PPD and PPA have a unique pattern of symptoms that may influence help-seeking differently. Thus, it is critical to examine both help-seeking intention and actual help-seeking behavior for each disorder group and comorbid PPD and PPA separately and compare them to women not clinically affected by PPD or PPA. In addition, research on PPA is still an emerging field [23], as previous studies have largely focused on PPD. Specifically, help-seeking for PPA is an understudied area of research, which this study will address. Additionally, the group not affected by clinical symptoms of PPD or PPA should be included as this group often reports subclinical symptoms, which can still impair postpartum women’s quality of life [24].

### Factors associated with help-seeking

**Fig. 1:**
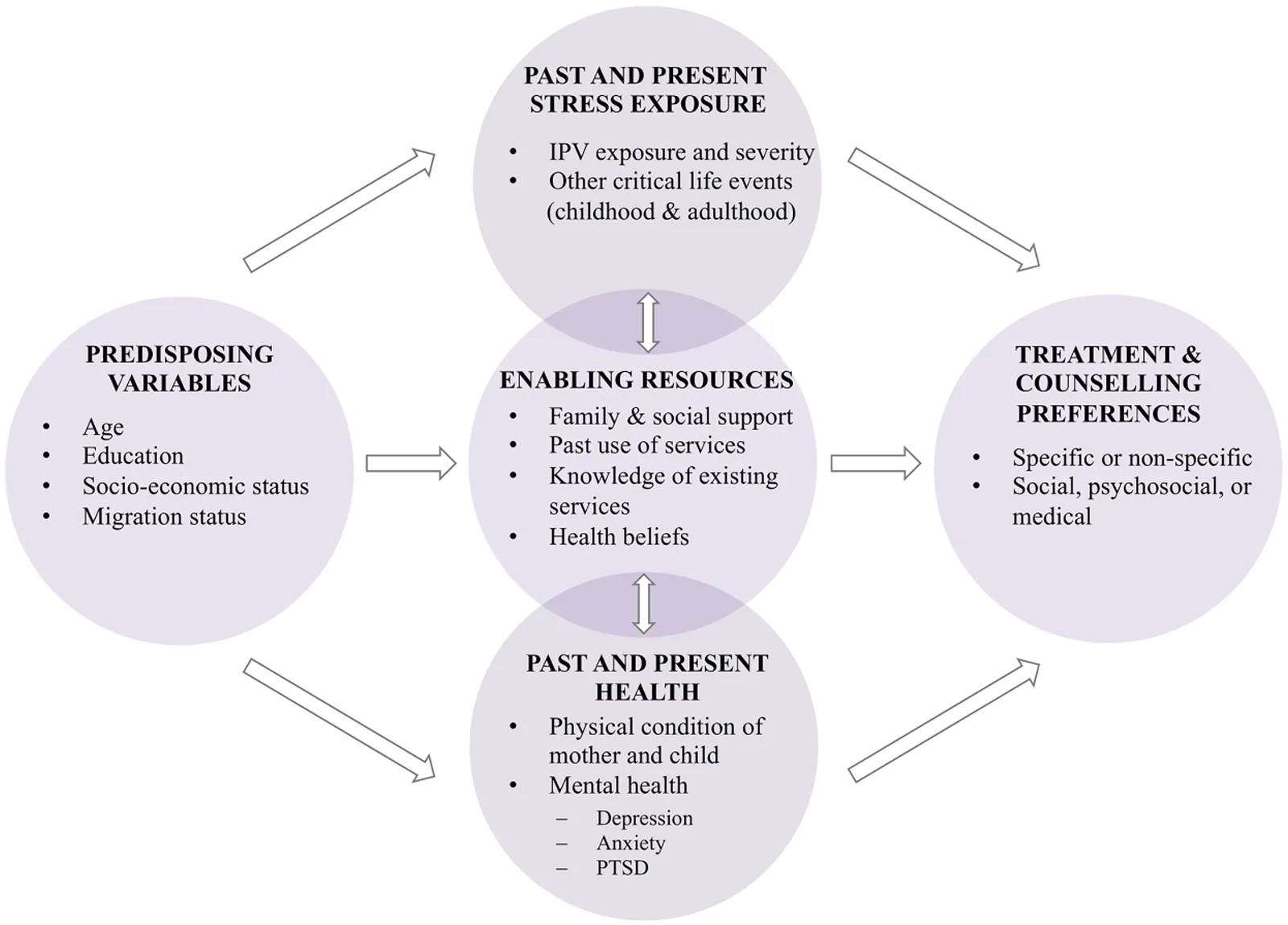
Theoretical model of help-seeking. Note. From preferences and barriers to counseling for and treatment of intimate partner violence, depression, anxiety, and posttraumatic stress disorder among postpartum women: study protocol of the cross-sectional study INVITE by Seefeld et al., 2022 [25]; (https://doi.org/doi: https://doi.org/10.3389/fpsyt.2022.836350), Reprinted with permission. Adapted From Andersen and Liang et al. [26, 27]

The theoretical framework of the INVITE (**IN**timate Partner **VI**olence care and **T**reatment pr**E**ferences in postpartum women) study by Seefeld and colleagues [25] based on models by Andersen [26] and Liang and colleagues [27] provides an overview of some potential factors associated with treatment and counseling preferences of postpartum women (Fig. 1) that are thought to be associated with service use and thus help-seeking (30; Fig. 1).

In the following, a selection of the potential associated factors found in the model are contextualized in current research and research gaps are highlighted. The first group of factors are the predisposing factors (Fig. 1). Actual help-seeking behavior among postpartum women has been found to be lower among those with a **migrant background.** Previous studies have suggested that this is due to higher barriers to help-seeking such as lack of social support and low income [28]. The negative effect of low **income** on help-seeking for PPD and/or PPA is suggested by some studies [28–30], while others could not find a clear association between income and help-seeking [31, 32]. The studies by Bina and colleagues [31] and Huang and colleagues [32] also found unclear results for **age** in relation to help-seeking, with one review including studies that found younger or older age to be negatively associated with help-seeking [31], and the other study finding no effect [32].

The second set of associated factors as seen in the model is the enabling resources. Postpartum women often view their symptoms of mental disorders as a phenomenon such as “baby blues” or “normal” worrying, which can inhibit help-seeking [22, 31, 33], suggesting that a lack of **recognition of a mental disorder** could affect help-seeking. Furthermore, research has shown that women who have **used** mental health **services in the past** [29, 31, 34], as well as those with greater **knowledge of** available mental health **services** [30–33] show more help-seeking. Positive attitudes toward seeking professional psychological help are associated with greater help-seeking intentions [30, 32], while negative **health beliefs**, such as a lack of belief in the efficacy of treatment act as barriers to help-seeking for PPD and PPA [31, 33]. A greater barrier to seeking help was found for mothers with comorbid PPD and PPA and negative health beliefs, compared to mothers with PPA and negative health beliefs alone [33]. Another enabling resource is **social support** which may serve as an associated factor with increased help-seeking [22, 28, 31, 35, 36]. Maguire and colleagues [33] found greater barriers to seeking professional help due to perceived lack of support for women with comorbid PPD and PPA than for women with PPA alone.

In the model category “past and present stress exposure”, experiencing **partner controlling behaviors** is found as a component of intimate partner violence (IPV; see Fig. 1). This factor may serve as a barrier to help-seeking, as women experiencing partner controlling behaviors tend to have poorer access to health care because the partner limits this access [37] and affected women are often socially isolated [37–39]. However, the effect of intimate partner controlling behaviors on help-seeking for PPD and PPA has not been examined yet.

Furthermore, a potential associated factor is **symptom severity**, included in the model category “past and present health”. In another publication of the INVITE study, higher symptom severity of PPD was associated with lower help-seeking intention for PPD, but this effect was reversed for PPA [40]. A meta-analysis showed that the relationship between symptom severity of PPD and actual help-seeking behavior was unclear [31], which is why this was further examined in the current study.

### The current study

As explained, there are still ambiguous findings and gaps in the literature and the relationship of the above-mentioned factors associated with professional help-seeking have not been sufficiently studied. Especially potential differences in these associations for women with symptoms of PPD, women with symptoms of PPA, women with symptoms of both PPD and PPA, and women without clinical symptoms of these disorders are not clear yet. Therefore, the current study aimed to investigate to what extent migrant background, income, age, recognition of mental disorder, knowledge of services, prior use of services, health beliefs, social support, partner controlling behaviors are associated with help-seeking intention among postpartum women with symptoms of PPD and/or PPA and women not clinically affected by PPD and/or PPA. Additionally, we examined whether these same factors as well as symptom severity are associated with actual help-seeking behavior among postpartum women with symptoms of PPD and/or PPA.

We hypothesized that postpartum women with a migrant background, lower income, a lack of recognition of their mental disorder, and those experiencing more partner controlling behaviors show lower, while women who have used mental health services before, know more mental health services, have higher social support, or hold more positive health beliefs show higher help-seeking. Furthermore, it was hypothesized that women with more severe symptoms of PPD show lower, while women with more severe symptoms of PPA show higher actual help-seeking behavior. The association between age and symptom severity for women with symptoms of both PPD and PPA with help-seeking was investigated in an exploratory manner.

## Methods

### Design

In the cross-sectional study **INVITE**, women living in and around Dresden, Germany, were recruited between November 2020 and February 2024 [25]. The study assessed symptoms of PPD, PPA, and (childbirth-related) posttraumatic stress disorder ((CB)-PTSD), as well as intimate partner violence (IPV) and related counseling and treatment preferences and barriers and facilitators to help-seeking. Recruitment was conducted by student assistants who mostly approached women in hospital maternity wards, at birth centers, and antenatal midwife appointments in Dresden. Women who gave birth to one or more children were contacted 10 weeks postpartum to schedule an approximately one-hour long telephone interview which then took place 3–4 months postpartum in German or English. Following the interview, all women were offered a list of possible treatment and counseling services for mental disorders and IPV. Participants received a monetary compensation of 20 € for participating in the study [25]. Data collection and management was facilitated using Research Electronic Data Capture (REDCap), a secure, web-based application for data capture as part of research studies, hosted at the “Koordinierungszentrum für Klinische Studien” at the Faculty of Medicine of the Technische Universität Dresden [41, 42]. This study involving human participants was reviewed and approved by the Ethics Committee of the Technische Universität Dresden (No: EK 139042016) to be conducted in accordance with the Declaration of Helsinki. All participants provided written informed consent to participate in this study.

### Sample

**Fig. 2:**
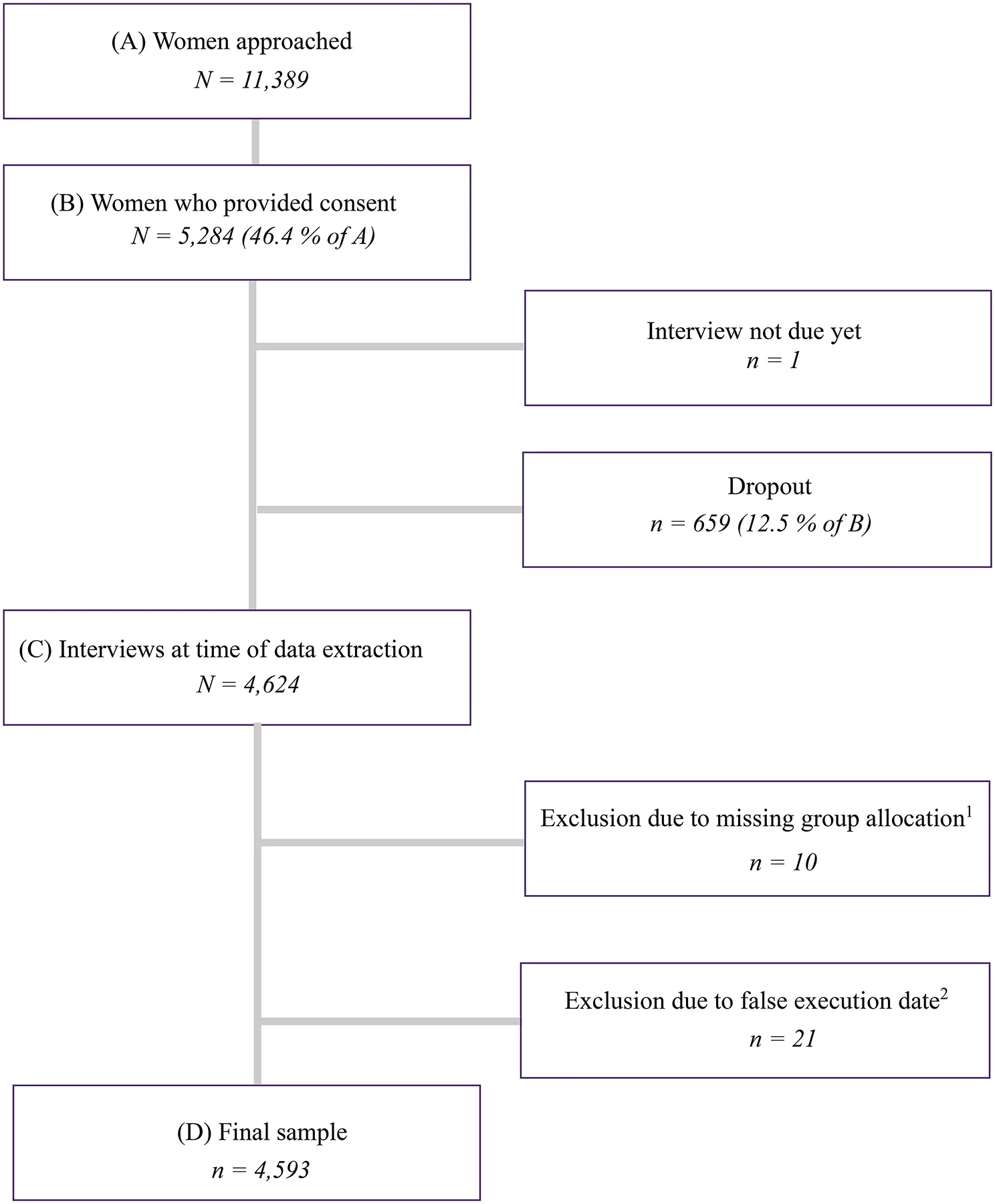
Flowchart of the current study. ^1^Women who did not complete the depression or anxiety screening were excluded, as they could not be assigned to a group (PPD, PPA, comorbid, not affected). ^2^Women interviewed either before 6 weeks or after 6 months postpartum were excluded

The current study is based on version 6 of the quality assured data files of the INVITE study (data extraction on 12/3/2024). A total of 11,389 women were approached and 46.4% provided written informed consent to participate in the study. A total of 659 participants either withdrew from the study or could no longer be reached, and one interview could not be completed within the planned time frame. Consequently, 4,624 participants finally took part in the study (Fig. 2).

Women were excluded from the study if they were interviewed earlier than 6 weeks or later than 6 months postpartum, as the planned time point for the study was 3–4 months postpartum and some variation was allowed [25]. Women who did not complete the screening for depression or anxiety were excluded because they could not be assigned to a group (PPD, PPA, comorbid, not affected by PPD or PPA). The final sample used in the current analyses was therefore *n* = 4,593 women and for the analysis of actual help-seeking behavior only the subsample affected by symptoms of PPD and/or PPA was used (*n* = 799).

## Materials

### Postpartum depression

The **Edinburgh Postnatal Depression Scale** (EPDS) is a self-report screening instrument that measures depressive symptoms over the past seven days [43] and was used to assess symptoms of PPD. The inventory consists of 10 items with different answer options measured on a 0–3 scale. A sum score was calculated ranging from 0 to 30 points, with higher scores indicating more severe symptoms of depression [43]. For group formation, the cut-off was set at ≥ 10 points, at which a woman was classified as having a probable depressive disorder [44]. Using this moderate cut-off value, a major depressive episode is detected in many cases but false positives are largely avoided [45, 46]. Internal consistency was good in the current sample (Cronbach’s α = 0.80).

### Postpartum anxiety

The anxiety subscale of the **Symptom Checklist-Revised** (SCL-90-R), a well-established self-report measure of psychiatric symptoms, was used to assess PPA [47]. It consists of 10 items asking for a range of possible anxiety symptoms. Women rated the severity of each symptom on a five-point scale from 0 to 4 (from *not at all* to *extremely*). Using the mean scores, T-scores were calculated with the normative table of the SCL-90-R and scores ≥ 60 were considered presenting a probable PPA [47]. Internal consistency was good in the current sample (Cronbach’s α = 0.82).

### Help-seeking

To assess **help-seeking intention**, all women were asked to indicate how likely they would be to seek help for postpartum mental disorders on an 11-point scale from very unlikely to very likely. This question was asked of both women with and without symptoms of PPD or PPA. Women without symptoms were asked to imagine the symptoms described and answer as if they were affected. This question was self-generated [25]. **Actual help-seeking behavior** was assessed by asking those women who self-reported having a current mental disorder whether they were currently receiving professional help for it. The question was adapted from another study collaborating with this research group [48]. For the analysis, a dichotomous variable was used to distinguish between those seeking help and those who did not. The variable was generated only for participants who screened positive for PPD and/ or PPA. The group who sought help but did not screen positive for PPD and/or PPA may include participants with symptoms of other mental disorders.

### Factors associated with help-seeking

Migrant background was operationalized as **first language other than German**. A person with a migrant background is defined as someone who was not born in Germany or whose parents or grandparents were not born in Germany [49]. First language is used because our study did not directly ask about the place of birth of the participants’ parents and grandparents to include both first and later generation immigrants in the study [50, 51]. A dichotomous variable, indicating whether a person had German as their first language or not, was computed.

**Income** was assessed by asking women directly about their household’s net monthly income [52], and **age** was calculated from the participants’ date of birth.

**Recognition of mental disorder** was assessed by asking women if they were currently suffering from a mental disorder and if yes, which one. These answers were compared with the screening results of PPD and PPA, and women were only classified as someone who recognized their mental disorder if they recognized their specific mental disorder (e.g., women in the PPD group had to recognize that they had PPD instead of PPA or another mental disorder). Women in the comorbid group (symptoms of PPD and PPA) only had to identify either depression or anxiety. This question was taken from the international survey of childbirth-related trauma (INTERSECT; 56).

To assess **knowledge of services**, women were presented with a range of treatment and counseling services for postpartum mental health difficulties and were asked to indicate for each service whether they already knew this service. A sum score was then calculated ranging from 0 to 13 indicating how many services the women knew. These questions were self-generated [25].

**Prior use of services** was assessed by directly asking participants, who reported having a mental disorder, whether they had sought professional or informal help for their mental disorder in the past or in the present, and a dichotomous variable was computed using this information. All types of services were included, such as psychotherapy, self-help groups, or help from family members. These questions were also self-generated [25].

**Health beliefs** were assessed using a 5-item subscale of a self-generated questionnaire on barriers to seeking treatment and counseling services based on the Health Belief Model [53]. For example, one item was “I think the benefits of counseling or treatment outweigh the disadvantages.”. Each item was rated on a 5-point Likert scale and a sum score was calculated ranging from 5 to 25. Higher scores indicated more negative health beliefs.

A short version of the Social Support Questionnaire (F-SozU) with 14 items rated on a 5-point scale was used to assess **social support** [54], and a mean score was calculated using all items. An example item was “I have a very close person whose help I can always count on.”.

**Partner controlling behaviors** were assessed using the Controlling Behavior subscale of the Violence Against Women Instrument, which comprises 7 items answered with “yes”, “no”, or “I don’t know”. Responses of “I don’t know” were counted as a missing value. An example item was “Thinking about your (current or most recent) husband/partner, would you say it is generally true that he/she expects you to ask his/her permission before seeking health care for yourself?”. A sum score between 0 and 6 was generated, indicating how many times a woman agreed with an item and thus how many different types of partner controlling behaviors she had experienced [55].

**Symptom severity** was assessed using the sum scores of the EPDS and the anxiety subscale of the SCL-90-R. For the comorbid group (symptoms of PPD and PPA), the two sum scores were z-standardized and then added together.

### Data analysis

Data were analyzed using SPSS Statistics software version 29. Prior to analysis, data were divided into four groups: women with symptoms of PPD, women with symptoms of PPA, women with symptoms of both PPD and PPA (comorbid), and women not affected by clinical levels of PPD or PPA, according to the cut-offs described in the Materials section. In Appendix A, all requirements for statistical analyses are listed.

First, multiple linear regression analyses (MLRs) were conducted with help-seeking intention as the outcome variable. The predictor variables were the expected associated factors migrant background, income, age, recognition of mental disorder, prior use of services, health beliefs, knowledge of services, social support, and partner controlling behaviors. Separate regression analyses were conducted for each group (PPD, PPA, comorbid, not affected by PPD or PPA) using bootstrapping with 1,000 iterations to address the issue of heteroscedasticity in the data. Only in the group not affected by PPD or PPA, recognition of mental disorder was not included as a predictor as it did not apply to this group.

For the outcome variable of actual help-seeking behavior, logistic regression analyses (LRs) were performed for the three relevant groups (PPD, PPA, comorbid). Only participants who screened positive for PPD and/or PPA were included, as our topic of interest was actual help-seeking behavior for these mental health disorders. The predictor variables were almost the same as for the MLRs, with the inclusion of symptom severity as a predictor, and prior use of services was not included in the analysis because it is a subset of the outcome and would therefore lead to an overestimation of the odds ratios (*ORs*). The metric predictors were z-standardized to obtain standardized and therefore interpretable *ORs*.

### Power analysis

An a priori power analysis was performed using G*Power version 3.1.9.6 [56]. All power analyses were calculated using a significance criterion of α = 0.05 and power = 0.80. For the multiple linear regression analyses, a sample size of *n* = 822 was required for a small effect size of *f*^*2*^ = 0.02. The smallest group of women with PPA (*n* = 88) was large enough to detect effect sizes in the medium to large range (*f²* = 0.21), the comorbid group (*n* = 203) and the PPD group (*n* = 508) were large enough to detect effect sizes in the small to medium range (comorbid *f²* = 0.08, PPD *f²* = 0.03), and the group not affected by PPD or PPA (*n = 3*,794) was large enough to detect small effect sizes (*f²* = 0.004).

For logistic regression, a sample size of *n* = 240 for each group was required for an *OR* = 1.68, which is considered a small effect [57]. The present sample sizes provided enough power to detect small effects (*OR* = 1.68) in the PPD group (*n* = 508) and effects in the small to medium range in the comorbid group (*OR* = 1.77; *n* = 203) and the PPA group (*OR* = 2.5; *n* = 88).

## Results

### Descriptive statistics

The final sample (*n* = 4,593) consisted of women with a mean age of 32.98 years (*SD* = 4.71). Most participants (92.1%) were native German speakers and had more than 10 years of education (74.2%). Almost all participants (97.7%) were in a couple relationship. Just over half of the sample (52.4%) were primiparous, while 47.6% were multiparous. Table 1 presents further descriptive statistics.

Table 2 shows the results of the correlation analysis of predictors and outcomes. PPD and PPA had a large statistically significant correlation (*r* = .629). Moderate correlations were found between recognition of mental disorder and PPD (*r* = .369) and PPA (*r* = .372), between health beliefs and help-seeking intention (*r* = .414), between actual help-seeking behavior and recognition of mental disorder (*r* = .409) and prior use of services (*r* = .373; 65). All other correlations were small [58] and can be found in Table 2.

**Table 1:** Descriptive statistics of the sample

Sample characteristics	Total sample^a^ (*n* = 4,593)
	*Mean ± SD (range) or n (%)*
**Maternal age** in years	32.98 ± 4.71 (16.00–53.96)
**Child´s age** in weeks	13.08 ± 2.71 (6.00–26.00)
**First language**	
German	4,226 (91.4%)
Other	396 (8.6%)
**Duration of residence in Germany** ^**b**^	
< 5 year	136 (3.0%)
5–10 years	135 (2.9%)
> 10 years	148 (3.2%)
Born in Germany	4,174 (90.9%)
**Income** ^c^	
< 1,250	102 (2.2%)
1,250–2,249	453 (9.9%)
2,250–2,999	574 (12.5%)
3,000–3,999	1,160 (25.4%)
4,000–4,999	1,147 (25.1%)
> 5,000	1,138 (24.9%)
**Other country of birth**	
Europe	148 (3.2%)
Outside of Europe	272 (5.9%)
**Partnership status**	
Partner	4,487 (97.7%)
No partner	106 (2.3%)
**Education**	
≤ 10 years	1,181 (25.7%)
> 10 years	3,409 (74.3%)
**Parity**	
Primiparous	2,408 (52.4%)
Multiparous	2,185 (47.6%)
2	1,680 (36.6%)
3	400 (8.7%)
4	81 (1.8%)
≥ 5	24 (0.5%)
**EPDS score**	5.54 ± 4.06 (0.00–27.00)
**SCL-90-R anxiety subscale**	2.54 ± 3.33 (0.00–36.00)
**Groups**	
Symptoms of PPD	508 (11.1%)
Symptoms of PPA	88 (1.9%)
Comorbid (Symptoms of PPD and PPA) Not clinically affected by PPD and PPA	203 (4.4%) 3,794 (82.6%)
**Help-seeking intention**	7.81 ± 2.14 (0.00–10.00)
Symptoms of PPD	7.66 ± 2.23 (0.00–10.00)
Symptoms of PPA	7.85 ± 2.82 (1.00–10.00)
Comorbid (Symptoms of PPD and PPA)	7.79 ± 2.41 (0.00–10.00)
Not clinically affected by PPD and PPA	7.84 ± 2.11 (0.00–10.00)
**Actual help-seeking behavior** ^**f**^	180 (3.9%)
Symptoms of PPD	49 (9.7%^d^)
Symptoms of PPA	12 (13.6%^d^)
Comorbid (Symptoms of PPD and PPA) Not clinically affected by PPD and PPA^e^	44 (21.7%^d^) 75 (2.0%^d^)
**Recognition of mental disorder**	128 (16.0%)
**Knowledge of services**	8.99 ± 2.77 (0.00–13.00)
**Prior use of services**	1,043 (22.7%)
**Health beliefs**	11.12 ± 2.77 (5.00–25.00)
**Social support**	4.42 ± 0.51 (1.07–5.00)
**Partner controlling behaviors**	0.28 ± 0.77 (0.00–6.00)

Note. EPDS = Edinburgh Postnatal Depression Scale, SCL-90-R = Symptom-Check-List-90-Revised (subscale anxiety), PPD = postpartum depression, PPA = postpartum anxiety. ^a^*n* varies slightly due to missing data from some participants, ^b^time since migration to Germany, ^c^in Euros per month and household, ^d^in relation to the respective symptom group, ^e^may include participants with symptoms other than PPD and/or PPA

**Table 2:** Pearson correlation matrix of all predictors and outcomes

	1.	2.	3.	4.	5.	6.	7.	8.	9.	10.	11.	12.	13.
1. EPDS	-												
2. SCL-90-R	0.629**	-											
3. Migrant background	0.111**	.058**	-										
4. Income	-0.113**	-0.090**	-0.041**	-									
5. Age	-0.082**	-0.072**	-0.012	0.211**	-								
6. Recognition of mental disorder	.369**	0.372**	0.009	-0.076**	-0.023	-							
7. Knowledge of services	-0.041**	0.012	-0.269**	0.006	0.008	0.057**	-						
8. Prior use of services	0.232**	0.266**	-0.035*	-0.086**	0.040**	0.227**	0.099**	-					
9. Health beliefs	0.144**	0.080**	0.054**	-0.097**	-0.124**	-0.008	-0.092**	0.136**	-				
10. Social support	-0.313**	-0.247**	-0.244**	0.175**	-0.034*	-0.171**	0.118**	-0.150**	-0.203**	-			
11. Partner controlling behaviors	0.164**	0.157**	0.047**	-0.175**	-0.064**	0.087**	0.013	0.110**	0.100**	-0.199**	-		
12. Help-seeking intention	-0.060**	0.009	0.032**	0.087**	0.072**	0.044**	0.092**	0.148**	-0.414**	0.189**	-0.057**	-	
13. Actual help-seeking behavior	0.230**	0.257**	-0.010	-0.065**	0.006	0.409**	0.063**	0.373**	-0.081**	-0.111**	0.080**	0.117**	-

Note. Statistically significant correlations are printed in bold. EPDS = Edinburgh Postnatal Depression Scale, SCL-90-R = Symptom-Check-List-90-Revised (subscale anxiety), *p < .05. **p < .01. ***p <.001

### Multiple Linear Regressions: Associations With Help-Seeking Intention

**Fig. 3:**
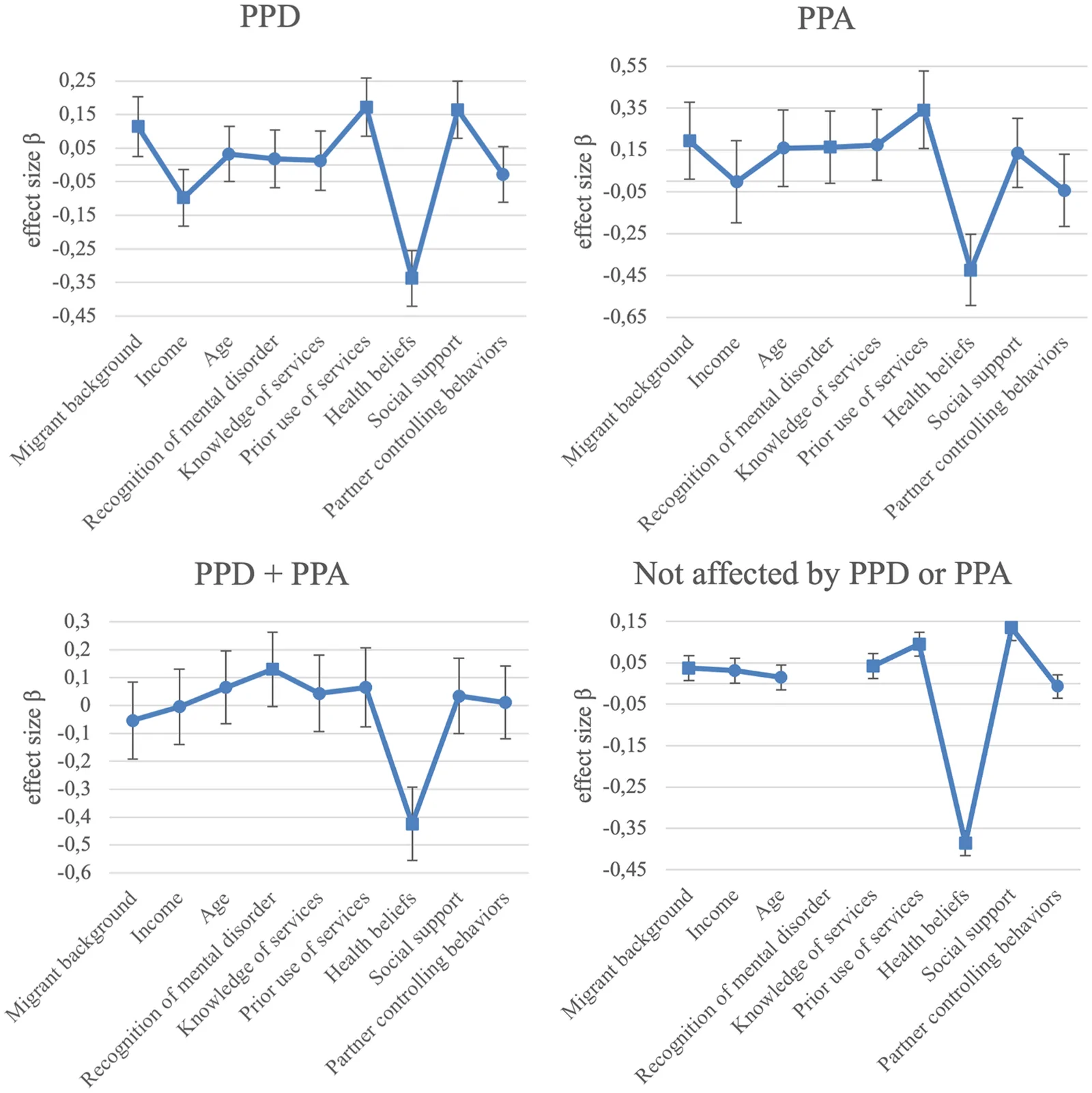
Results of the MLRs for each predictor in four groups. Note. PPD = symptoms of postpartum depression, PPA = symptoms of postpartum anxiety. Statistically significant effects are marked using blue squares

In each group (PPD, PPA, comorbid, not clinically affected by PPD or PPA), MLRs were calculated with help-seeking intention as the outcome variable. For each MLR, outliers in the outcome were excluded if they were more than three standard deviations away from the mean (PPD *n* = 5; PPA *n* = 1; comorbid *n* = 2; not clinically affected by PPD or PPA *n* = 31). All overall models were statistically significant (PPD, *F*[9] = 13.371, *p* < .001; PPA, *F*[9] = 7.772, *p* < .001; comorbid, *F*[9] = 7.354, *p* < .001; not clinically affected by PPD or PPA, *F*[8] = 128.815, *p* < .001). The model for the PPD group explained 19.0% of the variance in the criterion, the model for the PPA group 41.6%, the comorbid model 22.7%, and the model for the group not clinically affected by PPD and PPA explained 21.7%. The effects of the MLRs for each group are shown in Fig. 3 and the results are shown in Table 3.

**Table 3:** Results of the MLRs for the outcome help-seeking intention in four groups

Groups	PPD *n* = 503			PPA *n* = 87			comorbid (PPD+PPA) *n* = 201			Not clinically affected by PPD or PPA *n* = 3,763		
Independent Variables	β	95%-CI	*p*	β	95%-CI	*p*	β	95%-CI	*p*	β	95%-CI	*p*
Migrant background	0.114*	**[0.025; 0.203]**	0.010	0.194*	**[0.010; 0.378]**	0.042	-0.054	[-0.192; 0.084]	0.516	0.037*	**[0.007; 0.067]**	0.034
Income	-0.098*	**[-0.182; -0.014]**	0.027	-0.002	[-0.198; 0.194]	0.989	-0.004	[-0.139; 0.131]	0.940	0.031	[0.001; 0.061]	0.060
Age	0.032	[-0.050; 0.114]	0.468	0.159	[-0.024; 0.342]	0.072	0.065	[-0.065; 0.195]	0.319	0.015	[-0.015; 0.045]	0.357
Recognition of mental disorder	0.018	[-0.068; 0.104]	**[-0.009; 0.337]**	0.017	0.130*	**[-0.003; 0.263]**	0.030	-	-	-		
Knowledge of services	0.013	[-0.075; 0.101]	0.748	0.174	[0.005; 0.343]	0.098	0.043	[-0.094; 0.180]	0.544	0.042**	**[0.012; 0.072]**	0.008
Prior use of services	0.172***	**[0.085; 0.259]**	<0.001	0.342**	**[0.157; 0.527]**	0.002	0.065	[-0.077; 0.207]	0.418	0.095***	**[0.066; 0.124]**	<0.001
Health beliefs	-0.338***	**[-0.421; -0.255]**	<0.001	-0.423**	**[-0.594; -0.252]**	0.001	-0.424***	**[-0.556; -0.292]**	<0.001	-0.386***	**[-0.416; -0.356]**	<0.001
Social support	0.164**	**[0.079; 0.249]**	0.003	0.136	[-0.030; 0.302]	0.089	0.034	[-0.101; 0.169]	0.632	0.135***	**[0.104; 0.166]**	<0.001
Partner controlling behavior	-0.028	[-0.111; 0.055]	0.567	-0.043	[-0.216; 0.130]	0.591	0.011	[-0.120; 0.142]	0.852	-0.007	[-0.035; 0.021]	0.667
*R* ^*2*^	0.205			0.478			0.262			0.219		
*R* ^*2*^ *adjusted*	0.190			0.416			0.227			0.217		
*F(df)*	13.731*** (9)			7.722*** (9)			7.354*** (9)			128.815*** (8)		

Note. PPD = postpartum depression, PPA = postpartum anxiety. results are based on Bootstrapping *B* = 1000, statistically significant effects are printed in bold. *p < .05. **p < .01. ***p <.001

Within the MLRs, several statistically significant effects of predictors on help-seeking intention were found. In the PPD, PPA, and non-affected group having German as one’s first language was associated with increased help-seeking intention compared to having another first language, with the effect being strongest in the PPA group (β_PPD_ = 0.114; β_PPA_ = 0.194; β_non−affected_ = 0.037). In the PPD group, women with lower income served as a barrier to help-seeking intention (β = − 0.098). In the PPA group, recognition of PPA was associated with increased help-seeking intention (β = 0.164). In the group not clinically affected by PPD or PPA, knowing more services was associated with increased help-seeking intention (β = 0.042). Having ever used a service was associated with increased help-seeking intention in both the PPD and PPA groups, as well as in the group not clinically affected by PPD or PPA (β_PPD_ = 0.172; β_PPA_ = 0.342; β_non−affected_ = 0.095), with the largest effect in the PPA group. The strongest barrier was negative health beliefs in all groups (β_PPD_ = − 0.338; β_PPA_ = − 0.423; β_comorbid_ = − 0.305; β_non−affected_ = − 0.386). Additionally, in the PPD group, higher social support was associated with increased help-seeking intention (β = 0.164). The predictors age and partner controlling behaviors were not statistically significant in any group.

### Logistic regressions: associations with actual help-seeking behavior

In each symptom group (PPD, PPA, comorbid), LRs were calculated with actual help-seeking behavior as the outcome variable. Only the groups that included participants who screened positive for PPD and/or PPA were included in the analysis as we were interested in the actual help-seeking behavior for these mental health symptoms. Participants with missing values in at least one predictor variable were excluded in each LR due to listwise deletion (*n*_PPD_ = 14, *n*_PPA_ = 1, *n*_*PPD+PPA*_ = 5).

The LR model for PPD and the comorbid model were statistically significant (PPD χ²[9] = 99.210, *p* < .001, comorbid χ²[9] = 64.503, *p* < .001). The LR model for PPA was not statistically significant (χ²[9] = 11.614, *p* = .236). The model for PPD explained a small amount of variance (Nagelkerke’s *R*^2^ = 0.390) and the model for both PPD and PPA (comorbid) explained a moderate amount of variance (Nagelkerke’s *R*^2^ = 0.432; 66). For the PPD model, the overall classification accuracy was 91.3%, with a sensitivity of 34.0% and a specificity of 97.3%. For PPA, the overall classification accuracy was 86.2%, with a sensitivity of 16.7% and a specificity of 97.3%. In the comorbid model, the overall accuracy was 84.3%, with a sensitivity of 52.4% and a specificity of 92.9%. All three regression models were more reliable in predicting participants who did not seek help than those who did, which was the smaller proportion of the sample.

**Fig. 4:**
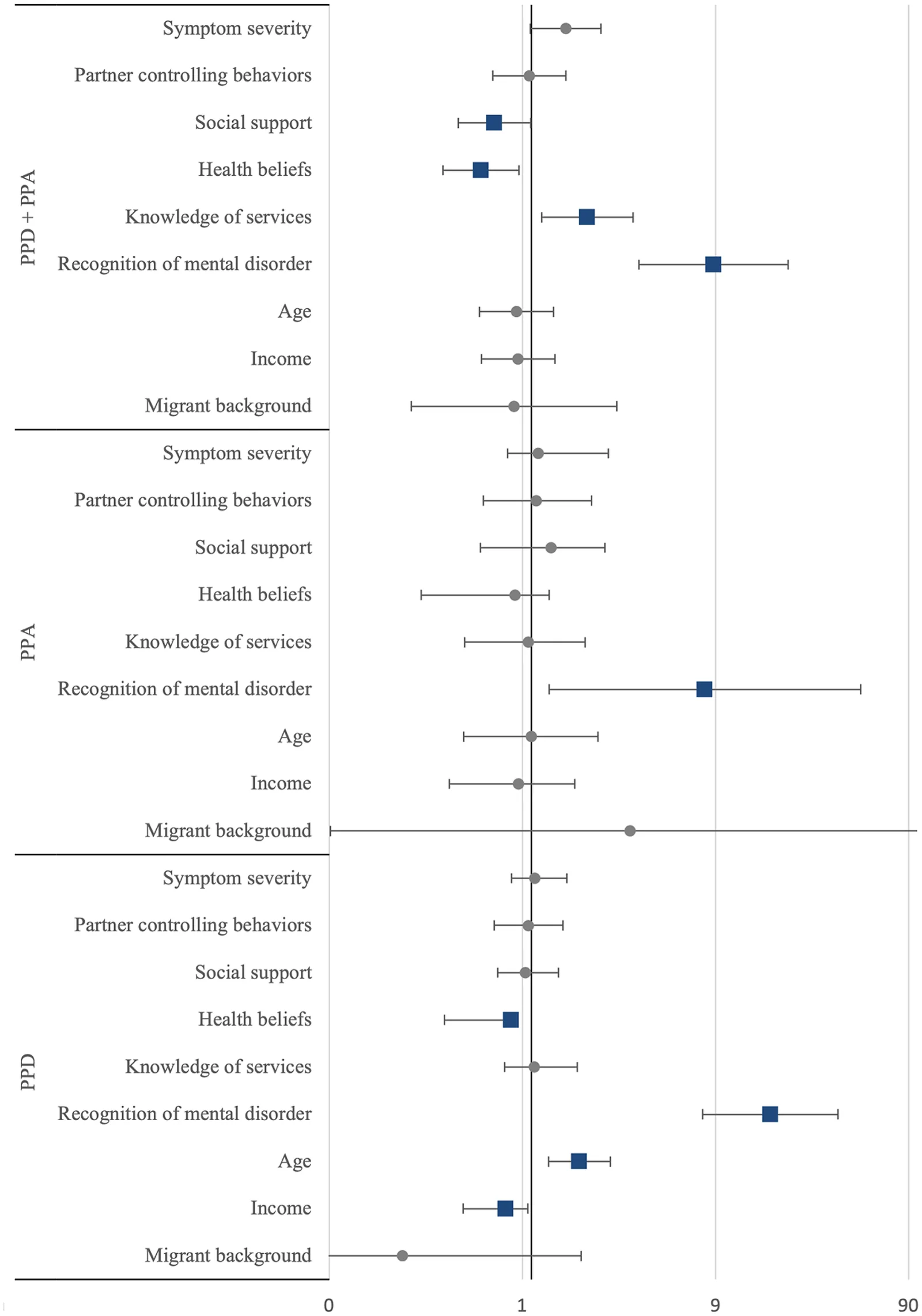
Results of the LRs for each predictor in the three groups. Note*.* PPD = postpartum depression, PPA = postpartum anxiety. Statistically significant effects are marked using blue squares

Statistically significant effects were found in all three models (see Table 4; Fig. 4). In the PPD group, lower income was a barrier to actual help-seeking behavior (*OR* = 0.653) and older age was associated with increased actual help-seeking behavior (*OR* = 1.777). Recognition of the mental disorder was associated with increased actual help-seeking behavior (*OR*_PPD_ = 17.283, *OR*_PPA_ = 7.928, *OR*_PPD + PPA_= 8.958; see Fig. 4). In the comorbid group, higher knowledge of services was associated with increased actual help-seeking behavior (*OR* = 1.943). More negative health beliefs were a barrier to actual help-seeking behavior in both the PPD (*OR* = 0.522) and the comorbid group (*OR* = 0.552). In the comorbid group, contrary to our hypothesis, lower social support was associated with increased actual help-seeking behavior (*OR* = 0.647; see Table 4). The predictors migrant background, partner controlling behaviors, and symptom severity were not statistically significant in any of the LR models.

**Table 4:** Results of the LRs for the outcome actual help-seeking behavior in three groups

Groups	PPD *n* = 493				PPA *n* = 87				comorbid (PPD+PPA) *n* = 198			
Independent Variables	OR	b (SE)	CI	*p*	OR	b (SE)	CI	*p*	OR	b (SE)	CI	*p*
Migrant background	0.215	-1.539 (1.089)	[0.025, 1.813]	0.158	3.250	1.179 (1.401)	[0.091, 115.918]	0.400	0.814	-0.206 (0.626)	[0.239, 2.771]	0.743
Income	0.653*	-0.427 (0.198)*	**[0.443**,** 0.962]**	0.031	0.797	-0.227 (0.380)	[0.377, 1.684]	0.550	0.856	-0.155 (0.225)	[0.552, 1.327]	0.490
Age	1.777**	0.575 (0.187)**	**[1.232**,** 2.563]**	0.002	0.995	-0.005 (0.411)	[0.446, 2.219]	0.991	0.839	-0.176 (0.225)	[0.540, 1.303]	0.434
Recognition of mental disorder	17.283***	2.850 (0.412)***	2.070 (0.924)*	**[1.236**,** 50.866]**	0.025	**8.789**	2.174 (0.453)***	**[3.616**,** 21.368]**	<0.001			
Knowledge of services	1.122	0.115 (0.220)	[0.729, 1.726]	0.600	0.927	-0.075 (0.364)	[0.452, 1.901]	0.836	1.948*	0.667 (0.278)*	**[1.130**,** 3.357]**	0.017
Health beliefs	0.522***	-0.651 (0.196)***	**[0.356**,** 0.765]**	<0.001	0.577	-0.551 (0.385)	[0.270, 1.234]	0.152	0.549*	-0.600 (0.233)*	**[0.348**,** 0.865]**	0.010
Social support	0.960	-0.040 (0.185)	[0.669, 1.380]	0.827	1.148	0.138 (0.384	[0.547, 2.409]	0.719	0.645*	-0.439 (0.222)*	**[0.418**,** 0.995]**	0.048
Partner controlling behaviors	0.967	-0.033 (0.208)	[0.643, 1.455]	0.872	1.077	0.074 (0.339)	[0.564, 2.058]	0.827	0.979	-0.021 (0.221)	[0.634, 1.510]	0.924
Symptom severity	1.102	0.097 (0.168)	[0.792, 1.533]	0.563	1.374	0.318 (0.299)	[0.753, 2.508]	0.288	1.509	0.412 (0.216)	[0.990, 2.300]	0.057
*Nagelkerke‘s R* ^*2*^	0.390					0.226			0.432			

Note. PPD = postpartum depression, PPA = postpartum anxiety. Metric predictors were z-standardized, statistically significant effects are printed in bold. *p < .05. ***p < .01. ***p <.001

## Discussion

The purpose of this study was to examine associated factors with help-seeking among postpartum women. In addition, we investigated how these factors differed between women with and without PPD and/or PPA. Negative health beliefs were found to represent the greatest barrier for help-seeking intention for all groups. Recognition of mental disorder was by far the most important factor associated with increased actual help-seeking behavior for all groups. While some associated factors could only be found for specific groups, for example, migrant background was associated with decreased help-seeking intention among women with PPD and PPA, and low income was associated with decreased help-seeking among women with PPD, other factors like partner controlling behaviors were not found to have any association with help-seeking.

Looking at the findings, we first discuss the “predisposing factors” of the theoretical model of help-seeking (Fig. 1). A woman’s **migrant background** was a barrier only for help-seeking intention in the PPD and in the PPA group, not for actual help-seeking behavior. Contrarily, another study found that women with a migrant background showed less actual help-seeking behavior postpartum [59]. Thus, the question of whether migrant background is a barrier to actual help-seeking behavior is not fully answered. A possible explanation for these ambiguous findings would be that migrant background is often associated with other factors like social support [28]. Low **income** was found to be a barrier to help-seeking intention only in the PPD group. Previous literature on the relationship between income and help-seeking is ambiguous [28, 29, 28, 32, 60], which could be due to low income in fact being a barrier specific to women with PPD. It has previously been suggested that mothers of low socioeconomic status should be specifically supported to seek help for postpartum mental health issues [61]. According to our results, this may be especially important for mothers with PPD. Older **age** was associated with increased actual help-seeking behavior only in the PPD group, demonstrating that age was unrelated to help-seeking intention and adding more clarity to previous findings on the relationship between age and help-seeking that were ambiguous in the direction of effects [31, 32].

In terms of “enabling resources” in the model of help-seeking, higher **recognition of mental disorder** seemed to be the most important factor associated with increased actual help-seeking behavior in all groups, notably the model for PPA was not statistically significant, though the predictor was, so this must be interpreted with caution. We found much smaller significant associations with help-seeking intention and only in the PPA and comorbid group. A possible explanation is that anxiety symptoms often include a need for reassurance due to worry [23], whereas depressive symptoms often lead to negative cognition and an inability to make decisions [6]. The findings show that the recognition of mental disorder can have a tremendous positive impact on actual help-seeking behavior. This finding is consistent with previous literature suggesting that many women do not recognize their postpartum mental disorder and show a lack of help-seeking [22, 31, 33, 35], with only 16.0% of those affected in this study recognizing their mental disorder and 13.1% of those affected actually seeking help. When interpreting this finding, it is important to consider the possibility that some of the women who did not participate or who dropped out were affected by a mental disorder and were seeking help for it.

Greater **knowledge of services** was associated with increased help-seeking intention among those not clinically affected by PPD or PPA and with actual help-seeking behavior in the comorbid group. Women who knew more services were already informed about where to get help for their postpartum mental disorder and might also have had more trust in the services [62]. Also relevant to those affected by either PPD or PPA could be the factor **prior use of services**, as those who had previously used services showed higher help-seeking intention. This lends further support to the need to familiarize women with available services [2]. We do not know how prior use of services would influence actual help-seeking behavior, as this variable was excluded from the analysis for statistical reasons.

Negative **health beliefs** emerged as one of the biggest associated factor for decreased help-seeking intention in all groups, with a smaller but still significant association with actual help-seeking behavior only in the PPD and comorbid group. These findings are in line with previous research showing that positive health beliefs are one of the most important factors associated with higher help-seeking for PPD and/or PPA [31, 33]. The large difference in effect sizes of health beliefs for help-seeking may be due to the similarity between health belief assessment and help-seeking intention as a more “cognitive” concept [63]. Both health beliefs and help-seeking intention are rooted in how individuals assess their needs and attitudes toward seeking help and therefore create a cognitive barrier.

While **social support** has been considered to be a facilitator for help-seeking among postpartum women in previous studies [22, 28, 31, 35, 36], our study suggests that this factor may only be associated with increased help-seeking intention for PPD and actual help-seeking behavior for women with symptoms of PPD or PPA. Strikingly, the association found for the comorbid group is the opposite of what we expected. Lower social support served as a factor associated with increased actual help-seeking behavior, perhaps because without the help of family and friends, seeking professional help may become more urgent, adding to an already existing urgency due to higher morbidity burden.

The model category “past and present stress exposure” included **partner controlling behaviors** as part of IPV. We found no association between partner controlling behaviors and postpartum help-seeking. In our study, the prevalence of experiencing partner controlling behavior related to health care seeking was low (1.0%), although the overall prevalence of partner controlling behavior in our study was not (17.2%). One explanation may be that this extreme form of partner controlling behavior is not very common and therefore this association could not be found.

Finally, no association of **symptom severity** categorized in past and present health (Fig. 1) and actual help-seeking behaviour was found. In another investigation of the present study sample an association between higher symptom severity and lower help-seeking intention for PPD, and the opposite for PPA was found [40]. Other research on symptom severity has suggested that higher symptom severity may be associated with higher actual help-seeking behavior for PPD [64], an association that is the opposite of our hypothesis based on the findings on help-seeking intention [40]. This calls for renewed consideration of how and why symptom severity may influence help-seeking.

To summarize, our findings highlight that there is a large difference in associated factors of help-seeking intentions and actual help-seeking behavior for symptoms of postpartum mental disorders, which has already been shown in the general population [65]. This gap may exist because intention is often shaped by factors, such as health beliefs [66], whereas actual help-seeking behavior requires overcoming other barriers [65–67], such as recognizing a mental disorder. However, it is important to emphasize that the two outcomes were investigated in two different populations: actual help-seeking behavior in women with symptoms of PPD and/or PPA, and help-seeking intention in women with and without symptoms. Each of the mental disorders we studied has unique characteristics and disorder-specific challenges. Women with PPD may have difficulty seeking help because of symptoms such as low energy, indecisiveness, or feelings of guilt and shame [6], which may interfere with help-seeking, and therefore may be more dependent on facilitators such as social support, older age, or higher income. In contrast, women with PPA often exhibit heightened arousal and hypervigilance [6], which may lead them to seek help out of a need for reassurance and control. For the comorbid group, the combined burden of PPD and PPA symptoms may lead to a greater sense of urgency to seek help. For them, higher levels of social support did not seem necessary or even helpful; rather, knowledge of mental health services or positive health beliefs seemed to facilitate seeking help. These group differences become apparent when actual help-seeking behavior is compared across groups. Descriptively, the prevalence of actual help-seeking in this study was lowest for PPD, higher for PPA, and highest for the comorbid group. However, group comparisons are limited by the fact that a different number of factors were included in each of the separate analyses and should be interpreted with this in mind.

### Strengths and limitations

This study is notable for its detailed exploration of the associated factors with help-seeking among postpartum women with symptoms of PPD and/or PPA. To our knowledge, it is the first study to examine both help-seeking intention and actual help-seeking behavior for symptoms of PPD and/or PPA, providing a more complete understanding of the postpartum help-seeking process. This, in turn, can improve access to appropriate services and identify groups at risk of not seeking help. In addition, examining specific groups, including those affected by comorbid conditions and those not clinically affected by PPD or PPA, allowed us to identify disorder-specific challenges associated with help-seeking. Moreover, the study’s recruitment method and the high response rate resulted in a large community sample. As the study was also available in English, it was easier to reach migrant women with a language barrier, and therefore the proportion of migrant women in our sample was almost the same as the average for the general population of Dresden between 2021 and 2023 [68–70]. Hence, compared to studies with less representative samples, more general conclusions can be drawn. The sample consisted of almost equal numbers of primi- and multiparous women, so that our results can be applied to both groups.

However, some limitations need to be considered. The study sample consisted of slightly older participants [71] with a slightly higher education than the average women giving birth in Saxony [72]. This means that there is a slight limitation in generalizing the results to the younger and less educated population of mothers giving birth in Dresden. While the results are specific to Dresden, they may offer insights for other regions with similar healthcare systems and socioeconomic conditions, particularly in other high-income country settings. A further limitation is that the gender of participants was not assessed, even though it is likely that not all individuals in the sample identified as women. Research has shown that 3–4% of people in Germany who were classified as female at birth, do not identify as women, with an even higher percentage among younger generations [73]. It is important to acknowledge the representation of other genders, such as non-binary and transgender people, in postpartum research.

Furthermore, the prevalence of PPA in our sample was quite small (1.9%) compared to the global prevalence [10, 11]. There is little reason to believe that the actual prevalence of PPA in our sample was significantly lower than the global rate, which suggests that the SCL-90-R [47] may not be the most appropriate tool for assessing postpartum populations, as it was only validated for this time period in a study with a very small sample size (*n* = 50; 76). The small sample size in the PPA group led to a lack of power, so that only moderate and large effects but not small effects could be found, meaning that the results must be interpreted with caution. Furthermore, the imbalance in group sizes hinders a comparison of the estimates. Also, as the overall LR model for PPA was not statistically significant, the significant predictor recognition of a mental disorder should be interpreted with caution. Furthermore, the model showed relatively low sensitivity, suggesting it was better at identifying women who did not seek help than those who did, which was due to the differences in group sizes.

Additionally, the cross-sectional design limits our ability to make causal inferences. A follow up would have been necessary to receive an overview of the trajectory of the variables and possible influences of participating in a study on help-seeking on these. Also, participants were only screened for symptoms of PPD and PPA, and they did not receive clinical diagnoses, which limits the interpretability of our findings. Furthermore, two of our main variables, help-seeking intention and actual help-seeking behavior consisted of only one self-generated item, therefore we cannot ensure sufficient reliability. When interpreting the findings on help-seeking intention, it must be noted that it was assessed in participants without symptoms of PPD and/or PPA. Participants were asked to imagine the symptoms described and therefore it should be clearly distinguished between those affected and those non-affected.

We found an unusually large OR of the effect of recognition of PPD on actual help-seeking behavior which could be due to quasi-complete separation, whereby a large share of the PPD group did not seek help and did not recognize PPD. To further support our hypothesis that recognizing a mental disorder is an important factor to increase help-seeking, more data of participants recognizing their PPD is needed.

Furthermore, some of the questionnaires used, namely service use, knowledge of services, health beliefs and help-seeking intention, were based on self-generated items, which limits empirical grounding and comparability. Furthermore, the reliance on self-reported data could introduce response biases, as participants might underreport or overreport symptoms of mental disorders or help-seeking due to social desirability or stigma associated with mental disorders.

### Research implications

There are several implications for future research that can be drawn from the present findings. Future studies should have an even more representative sample in terms of education and income to make potential associated factors with help-seeking for these groups more visible. Furthermore, longitudinal data would provide a clearer understanding of how help-seeking develops throughout the postpartum period. It is important to conduct this research with birthing people of different gender identities to identify potential differences in factors associated with help-seeking, as gender minorities may have negative experiences with care services, lowering their help-seeking [74]. The use of an instrument to assess PPA that is validated for the postpartum period is essential to improve findings in any future study about PPA.

### Practical implications

Our findings also suggest several practical implications. It is important to improve access to postpartum mental health services, as the prevalence of help-seeking can be considered low. This study has helped to identify specific groups at risk of low help-seeking, such as low-income mothers with PPD. These groups should be targeted to improve their help-seeking intention and actual help-seeking behavior. To improve recognition of mental disorders, which was the main associated factor of increased actual help-seeking behavior, screening of mothers for PPD [75] and PPA [76] should be included in routine postpartum health care. In addition, awareness campaigns that focus on improving mental health literacy [2] may be helpful for women’s ability to self-identify their postpartum mental disorder [21]. These campaigns should also include approaches to increase knowledge of services, as this is another barrier to help-seeking intention. This could be done by providing people with lists of services, an approach that was also used in our study [25]. Since one of the biggest factor associated with decreased help-seeking intention was negative health beliefs, addressing misconceptions about health care and personal beliefs about health, for example in postpartum (parenting) groups, may be helpful. All these measures could help to bridge the gap between help-seeking intention and actual help-seeking behavior, as this may require interventions that focus on cognitive barriers to help-seeking intention, while also focusing on awareness of postpartum mental disorders to increase actual help-seeking behavior.

## Conclusions

In conclusion, this study helped to identify factors associated with help-seeking among postpartum women with PPD and/or PPA, as well as those not clinically affected by it. The most important barrier to help-seeking intention were negative health beliefs, while recognition of mental disorder was the strongest associated factor for increased actual help-seeking behavior. In summary, we identified differences in factors associated with help-seeking and therefore disorder-specific challenges. In the future, a replication of these findings with more diverse samples should be strived for. In addition, the use of a better validated tool to assess PPA would strengthen the findings for PPA. Effectively, implementing routine mental disorder screenings for postpartum women [75, 76] and tailored postpartum mental health awareness campaigns [21, 77] are important to prevent a lack of help-seeking.

## Supplementary Information

Below is the link to the electronic supplementary material.

Supplementary Material 1

## Data Availability

The dataset presented in this article is not publicly available because of legal and ethical constraints. Public sharing of participant data was not included in the informed consent of the study. Requests to access the datasets should be directed to the project managers and principal investigators Susan Garthus-Niegel or Julia Schellong.

## Abbreviations

(CB)-PTSD: (Childbirth-related) posttraumatic stress disorder
DSM-5: Diagnostic and Statistical Manual of Mental Disorders fifth edition
EPDS: Edinburgh Postnatal Depression Scale
F-SozU: Social Support Questionnaire
GAD: Generalized anxiety disorder
INVITE: INtimate Partner VIolence care and Treatment prEferences in postpartum women
IPV: Intimate partner violence
LR: Logistic regression
MLR: Multiple linear regression
OR: Odds ratio
PPA: Postpartum anxiety
PPD: Postpartum depression
REDCap: Research Electronic Data Capture
SCL-90-R: Symptom Checklist-Revised

## References

[1] Zappas MP, Becker K, Walton-Moss B, Postpartum Anxiety. J Nurse Pract. 2021;17(1):60–4. 10.1016/j.nurpra.2020.08.017.

[2] Jones A. Postpartum Help-Seeking: The Role of Stigma and Mental Health Literacy. Matern Child Health J. 2022;26(5):1030–7. 10.1007/s10995-022-03399-1. 35258854

[3] Hahn-Holbrook J, Cornwell-Hinrichs T, Anaya I. Economic, health predictors of national postpartum depression prevalence: a systematic review, meta-analysis, and meta-regression of 291 studies from 56 countries. Front Psychiatry. 2018;8. 10.3389/fpsyt.2017.00248. PMC5799244 29449816

[4] Liu X, Wang S, Wang G. Prevalence and Risk Factors of Postpartum Depression in Women: A Systematic Review and Meta-analysis. J Clin Nurs. 2022;31(19–20):2665–77. 10.1111/jocn.16121. 34750904

[5] Zieß V, Seefeld L, Mojahed A, Martini J, Asselmann E, Schellong J, et al. Support preferences among women with and without postpartum depression and anxiety disorder. BMC Public Health. 2025;25(1):3048. 10.1186/s12889-025-24274-y. 40940653 PMC12427099

[6] American Psychiatric Association. Diagnostic and statistical manual of mental disorders [Internet]. 5th ed. American Psychiatric Association. 2013 [cited 2024 Feb 15]. Available from: https://psychiatryonline.org/doi/book/10.1176/appi.books.9780890425596.

[7] Romano M, Cacciatore A, Giordano R, La Rosa B. Postpartum period: three distinct but continuous phases. J Prenat Med. 2010;4(2):22–5. PubMed PMID: 22439056; PubMed Central PMCID: PMC3279173. 22439056 PMC3279173

[8] Meltzer-Brody S, Rubinow D. An overview of perinatal mood and anxiety disorders: epidemiology and etiology. In: Cox E, editor. Women’s Mood Disorders [Internet]. Cham: Springer International Publishing; 2021 [cited 2024 Feb 15]. p. 5–16. Available from: https://link.springer.com/10.1007/978-3-030-71497-0_2.

[9] Jordan V, Minikel M. Postpartum anxiety: More common than you think. J Fam Pract. 2019;68(3):165–165. 31039214

[10] Dennis CL, Falah-Hassani K, Shiri R. Prevalence of antenatal and postnatal anxiety: Systematic review and meta-analysis. Br J Psychiatry. 2017;210(5):315–23. 10.1192/bjp.bp.116.187179. 28302701

[11] Fawcett EJ, Fairbrother N, Cox ML, White IR, Fawcett JM. The prevalence of anxiety disorders during pregnancy and the postpartum period: a multivariate bayesian meta-analysis. J Clin Psychiatry. 2019;80(4). 10.4088/JCP.18r12527. PMC6839961 31347796

[12] Goodman JH, Watson GR, Stubbs B. Anxiety disorders in postpartum women: A systematic review and meta-analysis. J Affect Disord. 2016;203:292–331. 10.1016/j.jad.2016.05.033. 27317922

[13] Misri S, Abizadeh J, Sanders S, Swift E. Perinatal Generalized Anxiety Disorder: Assessment and Treatment. J Womens Health. 2015;24(9):762–70. 10.1089/jwh.2014.5150. PMC4589308 26125602

[14] Grigoriadis S, Graves L, Peer M, Mamisashvili L, Tomlinson G, Vigod SN, et al. A systematic review and meta-analysis of the effects of antenatal anxiety on postpartum outcomes. Arch Womens Ment Health. 2019;22(5):543–56. 10.1007/s00737-018-0930-2. 30523416

[15] Gentile S, Fusco ML. Untreated perinatal paternal depression: Effects on offspring. Psychiatry Res. 2017;252:325–32. 10.1016/j.psychres.2017.02.064. 28314228

[16] Slomian J, Honvo G, Emonts P, Reginster JY, Bruyère O. Consequences of maternal postpartum depression: A systematic review of maternal and infant outcomes. Womens Health. 2019;15:1745506519844044. 10.1177/1745506519844044. PMC6492376 31035856

[17] Letourneau N, Dennis CL, Benzies K, Duffett-Leger L, Stewart M, Tryphonopoulos PD, et al. Postpartum Depression is a Family Affair: Addressing the Impact on Mothers, Fathers, and Children. Issues Ment Health Nurs. 2012;33(7):445–57. 10.3109/01612840.2012.673054. 22757597

[18] Duan Z, Wang Y, Jiang P, Wilson A, Guo Y, Lv Y, et al. Postpartum depression in mothers and fathers: a structural equation model. BMC Pregnancy Childbirth. 2020;20(1):537. 10.1186/s12884-020-03228-9. 32933502 PMC7493423

[19] Letourneau N, Leung B, Ntanda H, Dewey D, Deane AJ, Giesbrecht GF, et al. Maternal and paternal perinatal depressive symptoms associate with 2- and 3-year-old children’s behaviour: findings from the APrON longitudinal study. BMC Pediatr. 2019;19(1):435. 10.1186/s12887-019-1775-1. 31722682 PMC6852959

[20] Cacciola E, Psouni E. Insecure Attachment and Other Help-Seeking Barriers among Women Depressed Postpartum. Int J Environ Res Public Health. 2020;17(11):3887. 10.3390/ijerph17113887. 32486285 PMC7313466

[21] Daehn D, Rudolf S, Pawils S, Renneberg B. Perinatal mental health literacy: knowledge, attitudes, and help-seeking among perinatal women and the public – a systematic review. BMC Pregnancy Childbirth. 2022;22(1):574. 10.1186/s12884-022-04865-y. 35854232 PMC9295513

[22] Hu Y, Huang S, Xiao M, Fu B, Tang G, Lommel L, et al. Barriers and facilitators of psychological help-seeking behaviors for perinatal women with depressive symptoms: A qualitative systematic review based on the Consolidated Framework for Implementation Research. Midwifery. 2023;122:103686. 10.1016/j.midw.2023.103686. 37119670

[23] Feldman N, Hibara A, Ye J, Macaranas A, Larkin P, Hendrix E, et al. Postpartum anxiety: a state-of-the-art review. Lancet Psychiatry. 2025;12(12):947–59. 10.1016/S2215-0366. (25)00197-X PubMed PMID: 40907501. 40907501

[24] Meaney MJ. Perinatal Maternal Depressive Symptoms as an Issue for Population Health. Am J Psychiatry. 2018;175(11):1084–93. 10.1176/appi.ajp.2018.17091031. 30068258

[25] Seefeld L, Mojahed A, Thiel F, Schellong J, Garthus-Niegel S. Preferences and Barriers to Counseling for and Treatment of Intimate Partner Violence, Depression, Anxiety, and Posttraumatic Stress Disorder Among Postpartum Women: Study Protocol of the Cross-Sectional Study INVITE. Front Psychiatry. 2022;13:836350. 10.3389/fpsyt.2022.836350. 35422719 PMC9001846

[26] Andersen RM. Revisiting the Behavioral Model and Access to Medical Care: Does it Matter? J Health Soc Behav. 1995;36(1):1–10. 10.2307/2137284. 7738325

[27] Liang B, Goodman L, Tummala-Narra P, Weintraub S. A Theoretical Framework for Understanding Help-Seeking Processes Among Survivors of Intimate Partner Violence. Am J Community Psychol. 2005;36(1–2):71–84. 10.1007/s10464-005-6233-6. 16134045

[28] Webb R, Ford E, Shakespeare J, Easter A, Alderdice F, Holly J, et al. Conceptual framework on barriers and facilitators to implementing perinatal mental health care and treatment for women: the MATRIx evidence synthesis. Health Soc Care Deliv Res. 2024;1–187. 10.3310/KQFE0107. 38317290

[29] Manso-Córdoba S, Pickering S, Ortega MA, Asúnsolo Á, Romero D. Factors Related to Seeking Help for Postpartum Depression: A Secondary Analysis of New York City PRAMS Data. Int J Environ Res Public Health. 2020;17(24):9328. 10.3390/ijerph17249328. 33322171 PMC7763494

[30] Deng C, Wang X, Du X, Xiao Y, Yan B, Li Y, et al. Awareness, Attitudes, and Help-Seeking Intention Towards Perinatal Depression Among Women from Different Ethnic Groups in Western Rural China. Int J Womens Health. 2026;18:1–14. 10.2147/IJWH.S600790. PMC13155397 42111333

[31] Bina R. Predictors of postpartum depression service use: A theory-informed, integrative systematic review. Women Birth. 2020;33(1):e24–32. 10.1016/j.wombi.2019.01.006. 30718105

[32] Huang S, Hu Y, Fu B, Tang G, Chen Z, Zhang L, et al. Help-Seeking Intentions for Depression and Associated Factors among Chinese Perinatal Women: A Cross-Sectional Study. Int J Environ Res Public Health. 2023;20(3):2288. 10.3390/ijerph20032288. 36767654 PMC9916212

[33] Maguire PN, Bhullar N, Cosh SM, Wootton BM. Help-seeking and treatment delivery preferences for women experiencing perinatal anxiety symptoms. Behav Cogn Psychother. 2023;51(4):271–85. 10.1017/S1352465823000012. 37009749

[34] Ride J, Lancsar E. Women’s Preferences for Treatment of Perinatal Depression and Anxiety: A Discrete Choice Experiment. PLoS ONE. 2016;11(6):e0156629. 10.1371/journal.pone.0156629. 27258096 PMC4892671

[35] Harrison V, Moore D, Lazard L. Supporting perinatal anxiety in the digital age; a qualitative exploration of stressors and support strategies. BMC Pregnancy Childbirth. 2020;20(1):363. 10.1186/s12884-020-02990-0. 32546131 PMC7298791

[36] Sorsa MA, Kylmä J, Bondas TE. Contemplating Help-Seeking in Perinatal Psychological Distress—A Meta-Ethnography. Int J Environ Res Public Health. 2021;18(10):5226. 10.3390/ijerph18105226. 34069073 PMC8156805

[37] Baird K, Sapkota D. Domestic and Family Violence in Pregnancy and the Postpartum Period. In: Martin CR, Preedy VR, Patel VB, editors. Handbook of Anger, Aggression, and Violence [Internet]. Cham: Springer International Publishing; 2023 [cited 2024 Feb 29]. pp. 1063–83. Available from: https://link.springer.com/10.1007/978-3-031-31547-3_59.

[38] Boxall H, Morgan A. Experiences of coercive control among Australian women [Internet]. Australian Institute of Criminology; 2021 [cited 2024 Feb 27]. Available from: https://www.aic.gov.au/publications/sb/sb30.

[39] Wydall S, Zerk R. Listen to me, his behaviour is erratic and I’m really worried for our safety. ’: Help-seeking in the context of coercive control. Criminol Crim Justice. 2021;21(5):614–32. 10.1177/1748895819898513.

[40] Seefeld L, Jehn V, Schellong J, Garthus-Niegel S. Access and barriers to treatment and counseling for postpartum women with and without symptoms of (CB-)PTSD within the cross-sectional study INVITE. BMC Pregnancy Childbirth. 2026;26(1):93. 10.1186/s12884-026-08660-x. 41578202 PMC12849266

[41] Harris PA, Taylor R, Thielke R, Payne J, Gonzalez N, Conde JG. Research electronic data capture (REDCap)—A metadata-driven methodology and workflow process for providing translational research informatics support. J Biomed Inf. 2009;42(2):377–81. 10.1016/j.jbi.2008.08.010. PMC2700030 18929686

[42] Harris PA, Taylor R, Minor BL, Elliott V, Fernandez M, O’Neal L, et al. The REDCap consortium: Building an international community of software platform partners. J Biomed Inf. 2019;95:103208. 10.1016/j.jbi.2019.103208. PMC7254481 31078660

[43] Cox JL, Holden JM, Sagovsky R. Detection of Postnatal Depression: Development of the 10-item Edinburgh Postnatal Depression Scale. Br J Psychiatry. 1987;150(6):782–6. 10.1192/bjp.150.6.782. 3651732

[44] Liu Y, Guo N, Li T, Zhuang W, Jiang H. Prevalence and Associated Factors of Postpartum Anxiety and Depression Symptoms Among Women in Shanghai, China. J Affect Disord. 2020;274:848–56. 10.1016/j.jad.2020.05.028. 32664024

[45] Gibson J, McKenzie-McHarg K, Shakespeare J, Price J, Gray R. A systematic review of studies validating the Edinburgh Postnatal Depression Scale in antepartum and postpartum women. Acta Psychiatr Scand. 2009;119(5):350–64. 10.1111/j.1600-0447.2009.01363.x. 19298573

[46] Lagerberg D, Magnusson M, Sundelin C. Drawing the line in the Edinburgh Postnatal Depression Scale (EPDS): a vital decision [Internet]. 2011;23(1). 10.1515/ijamh.2011.005. 21721360

[47] Franke G. Die symptom-Checkliste von Derogatis (SCL-90-R) - Deutsche Version - Manual. 2002.

[48] INTERSECT. Study Registration of the International Survey of Childbirth-Related Trauma (INTERSECT). Available online at: https://www.researchregistry.com/browse-the-registry#home/registrationdetails/5ffc7453702012001b80a58c/. Accessed at February 29, 2024. 2021; Available online at: https://www.researchregistry.com/browse-the-registry#home/registrationdetails/5ffc7453702012001b80a58c/. Accessed at February 29, 2024.

[49] Salentin K. Sampling the Ethnic Minority Population in Germany. The Background to Migration Background. methods. data. 2014;28:Pages. 10.12758/MDA.2014.002.

[50] German National Cohort Consortium. The German National Cohort: aims, study design and organization. Eur J Epidemiol. 2014;29(5):371–82. 10.1007/s10654-014-9890-7. 24840228 PMC4050302

[51] Kress V, Steudte-Schmiedgen S, Kopp M, Förster A, Altus C, Schier C, et al. The Impact of Parental Role Distributions, Work Participation, and Stress Factors on Family Health-Related Outcomes: Study Protocol of the Prospective Multi-Method Cohort Dresden Study on Parenting, Work, and Mental Health (DREAM). Front Psychol. 2019;10:1273. 10.3389/fpsyg.2019.01273. 31263435 PMC6584823

[52] Lampert T, Kroll LE. Die Messung des sozioökonomischen Status in sozialepidemiologischen Studien. In: Richter M, Hurrelmann K, editors. Gesundheitliche Ungleichheit: Grundlagen, Probleme, Perspektiven [Internet]. Wiesbaden: VS Verlag für Sozialwissenschaften; 2009 [cited 2024 Feb 26]. p. 309–34. Available from: 10.1007/978-3-531-91643-9_18.

[53] Rosenstock IM. The Health Belief Model and Preventive Health Behavior. Health Educ Monogr. 1974;2(4):354–86. 10.1177/109019817400200405.

[54] Fydrich T, Sommer G, Tydecks S, Brähler E. Fragebogen zur sozialen Unterstützung (F-SozU): Normierung der Kurzform (K-14. Z Für Med Psychol. 2009;18:43–8.

[55] Ellsberg M, Heise L, Geneva. Switzerland: Washington, DC: World Health Organization; Program for Appropriate Technology in Health (PATH); 2005. 257 p.

[56] Faul F, Erdfelder E, Lang AG, Buchner A. G*Power 3: A flexible statistical power analysis program for the social, behavioral, and biomedical sciences. Behav Res Methods. 2007;39(2):175–91. 10.3758/BF03193146. 17695343

[57] Chen H, Cohen P, Chen S. How big is a big odds ratio? Interpreting the magnitudes of odds ratios in epidemiological studies. Commun Stat Comput. 2010 Mar 31. 10.1080/03610911003650383.

[58] Cohen J. Statistical Power Analysis for the Behavioral Sciences. 2nd ed. New York: Routledge; 1988. p. 567. 10.4324/9780203771587.

[59] Santiá P, De Montgomery CJ, Pedersen TP, Marti-castaner M. Differences in postpartum mental healthcare among women with identified needs: the role of migration status. Scand J Public Health. 2023 Oct 13;14034948231178337. 10.1177/14034948231178337. 37837218

[60] Deng C, Yan B, Du X, Xiao Y, Li Y, Luo S, et al. Awareness, attitudes, and help-seeking intention towards perinatal depression among women from different ethnic groups in Western Rural China [preprint] [Internet]. In Review; 2023 Sep [cited 2024 Feb 16]. Available from: https://www.researchsquare.com/article/rs-3230563/v1. 10.2147/IJWH.S600790 PMC13155397 42111333

[61] Shorey S, Ng JQX, Liu VC, Chan YH, Chee CYI. Nurturing networks: A prospective longitudinal study of factors influencing help-seeking behaviours among mothers from low socioeconomic status. J Adv Nurs. 2024;n/a(n/a). 10.1111/jan.16382. 39136177

[62] Gaebel W, Muijen M, Baumann AE, Bhugra D, Wasserman D, van der Gaag RJ, et al. EPA Guidance on Building Trust in Mental Health Services. Eur Psychiatry. 2014;29(2):83–100. 10.1016/j.eurpsy.2014.01.001. 24506936

[63] Alyafei A, Easton-Carr R. The health belief model of behavior change. In: StatPearls [Internet]. Treasure Island (FL): StatPearls Publishing; 2024 [cited 2024 Nov 4]. Available from: http://www.ncbi.nlm.nih.gov/books/NBK606120/. PubMed PMID: 39163427. 39163427

[64] Jones A, Duong CC. Exploring predictors of help-seeking behaviors among women with postpartum depression: A social media recruited sample. Public Health Nurs. 2024;41(5):894–900. 10.1111/phn.13350. 38853282

[65] Tomczyk S, Schomerus G, Stolzenburg S, Muehlan H, Schmidt S, Ready. Willing and Able? An Investigation of the Theory of Planned Behaviour in Help-Seeking for a Community Sample with Current Untreated Depressive Symptoms. Prev Sci. 2020;21(6):749–60. 10.1007/s11121-020-01099-2. 32140825 PMC7366606

[66] Langley EL, Wootton BM, Grieve R. The Utility of the Health Belief Model Variables in Predicting Help-Seeking Intention for Anxiety Disorders. Aust Psychol. 2018;53(4):291–301. 10.1111/ap.12334.

[67] Li W, Denson LA, Dorstyn DS. Understanding Australian university students’ mental health help-seeking: An empirical and theoretical investigation. Aust J Psychol. 2018;70(1):30–40. 10.1111/ajpy.12157.

[68] Kommunale Statistikstelle Dresden. Dresden in Zahlen I. Quartal 2022 [Internet]. 2022 [cited 2024 Oct 6]. Available from: https://www.dresden.de/media/pdf/statistik/Dresden-in-Zahlen_2023_II_Quartal.pdf.

[69] Kommunale Statistikstelle Dresden. Dresden in Zahlen II. Quartal 2023. 2023.

[70] Kommunale Statistikstelle Dresden. Dresden in Zahlen I. Quartal 2024. 2024.

[71] Statistisches Landesamt Sachsen. Geburten - Statistik - sachsen.de [Internet]. 2024 [cited 2024 Oct 6]. Available from: https://www.statistik.sachsen.de/html/geburten.html.

[72] Statistisches Landesamt Sachsen. Bildung und Erwerbstätigkeit - Statistik - sachsen.de [Internet]. 2024 [cited 2024 Nov 1]. Available from: https://www.statistik.sachsen.de/html/statistischbetrachtet-familie-bildung-und-erwerbstaetigkeit.html.

[73] Ipsos. LGBT+ Pride 2021 Global survey points to a generation gap around gender identity and sexual attraction | Ipsos [Internet]. 2021 [cited 2024 Feb 26]. Available from: https://www.ipsos.com/en/lgbt-pride-2021-global-survey-points-generation-gap-around-gender-identity-and-sexual-attraction.

[74] Klittmark S, Malmquist A, Karlsson G, Ulfsdotter A, Grundström H, Nieminen K. When complications arise during birth: LBTQ people’s experiences of care. Midwifery. 2023;121:103649. 10.1016/j.midw.2023.103649. 37003045

[75] Lang E, Colquhoun H, LeBlanc JC, Riva JJ, Moore A, Traversy G, et al. Recommendation on instrument-based screening for depression during pregnancy and the postpartum period. CMAJ. 2022;194(28):E981–9. 10.1503/cmaj.220290. PubMed PMID: 35878894. 35878894 PMC9328462

[76] Murthy S, Haeusslein L, Bent S, Fitelson E, Franck LS, Mangurian C. Feasibility of universal screening for postpartum mood and anxiety disorders among caregivers of infants hospitalized in NICUs: a systematic review. J Perinatol. 2021;41(8):1811–24. 10.1038/s41372-021-01005-w. 33692474 PMC8349842

[77] Grissette B. An educational intervention addressing postpartum depression and help-seeking behavior: a pilot study. Nurs Diss PhD. 2022 Mar 31. 10.31922/hjka-fy80.

