# Supplementary material for: The silent struggle after childbirth – barriers and facilitators to help-seeking for postpartum depression and anxiety
Source: BMC Psychiatry. 2026 Aug 17;26:742. doi: 10.1186/s12888-026-08509-9 (PMC13599066; doi:10.1186/s12888-026-08509-9)
Supplement: Supplementary file 1 [file 12888_2026_8509_MOESM1_ESM.docx]

# **Appendix A: Requirements of all Statistical Analyses**

To get an overview of the data, descriptive statistics were calculated for all relevant variables, and the mean and standard deviation were calculated for each variable. The data were tested for the assumptions of both multiple linear regression and logistic regression. For the MLR, first, the presence of a linear relationship between the independent and dependent variables was tested. Second, outliers in the outcome variable were identified, with values in the outcome having studentized residuals below -3 or above +3, and excluded. Third, the independence of the residuals was tested to ensure that there was no autocorrelation. Fourth, multicollinearity among predictors was tested to ensure that the independent variables were not too highly correlated. Fifth, homoscedasticity was tested to confirm that the residuals had constant variance across all levels of the predictors. Finally, the normality of the residuals was assessed. For the LR, a linear relationship between the predictors and the log odds of the outcome was tested, multicollinearity among the predictors was assessed, and outliers were identified. The results of the assumption testing, including the number of excluded variables, for both MLR and LR are reported in the Results section.
